# Microbial and Viral Communities and Their Antibiotic Resistance Genes Throughout a Hospital Wastewater Treatment System

**DOI:** 10.3389/fmicb.2020.00153

**Published:** 2020-02-19

**Authors:** Morgan L. Petrovich, Adi Zilberman, Aviv Kaplan, Gefen R. Eliraz, Yubo Wang, Kathryn Langenfeld, Melissa Duhaime, Krista Wigginton, Rachel Poretsky, Dror Avisar, George F. Wells

**Affiliations:** ^1^Department of Civil and Environmental Engineering, Northwestern University, Evanston, IL, United States; ^2^The Water Research Center, School of The Environment and Earth Sciences, Faculty of Exact Sciences, Tel Aviv University, Tel Aviv, Israel; ^3^Department of Civil and Environmental Engineering, University of Michigan, Ann Arbor, MI, United States; ^4^Department of Ecology and Evolutionary Biology, University of Michigan, Ann Arbor, MI, United States; ^5^Department of Biological Sciences, The University of Illinois at Chicago, Chicago, IL, United States

**Keywords:** wastewater treatment, antibiotic resistance, virus, hospital wastewater, metagenomics

## Abstract

Antibiotic resistance poses a serious threat to global public health, and antibiotic resistance determinants can enter natural aquatic systems through discharge of wastewater effluents. Hospital wastewater in particular is expected to contain high abundances of antibiotic resistance genes (ARGs) compared to municipal wastewater because it contains human enteric bacteria that may include antibiotic-resistant organisms originating from hospital patients, and can also have high concentrations of antibiotics and antimicrobials relative to municipal wastewater. Viruses also play an important role in wastewater treatment systems since they can influence the bacterial community composition through killing bacteria, facilitating transduction of genetic material between organisms, and modifying the chromosomal content of bacteria as prophages. However, little is known about the fate and connections between ARGs, viruses, and their associated bacteria in hospital wastewater systems. To address this knowledge gap, we characterized the composition and persistence of ARGs, dsDNA viruses, and bacteria from influent to effluent in a pilot-scale hospital wastewater treatment system in Israel using shotgun metagenomics. Results showed that ARGs, including genes conferring resistance to antibiotics of high clinical relevance, were detected in all sampling locations throughout the pilot-scale system, with only 16% overall depletion of ARGs per genome equivalent between influent and effluent. The most common classes of ARGs detected throughout the system conferred resistance to aminoglycoside, cephalosporin, macrolide, penam, and tetracycline antibiotics. A greater proportion of total ARGs were associated with plasmid-associated genes in effluent compared to in influent. No strong associations between viral sequences and ARGs were identified in viral metagenomes from the system, suggesting that phage may not be a significant vector for ARG transfer in this system. The majority of viruses in the pilot-scale system belonged to the families *Myoviridae*, *Podoviridae*, and *Siphoviridae*. Gammaproteobacteria was the dominant class of bacteria harboring ARGs and the most common putative viral host in all samples, followed by Bacilli and Betaproteobacteria. In the total bacterial community, the dominant class was Betaproteobacteria for each sample. Overall, we found that a variety of different types of ARGs and viruses were persistent throughout this hospital wastewater treatment system, which can be released to the environment through effluent discharge.

## Introduction

Wastewater and wastewater treatment plants (WWTPs) contain a variety of antibiotic resistance genes (ARGs) that can be transmitted into the environment through the discharge of effluents (Szczepanowski et al., 2009; Berendonk et al., 2015; Chu et al., 2018; Petrovich et al., 2018). Hospital wastewater is thought to be a particularly important driver of selection for ARGs in WWTPs because it often contains antibiotic resistant bacteria and antibiotic residues at higher concentrations than other urban wastewater sources (Baquero et al., 2008; Rodriguez-Mozaz et al., 2015). Since hospital wastewater also contains a mixture of chemicals such as antibiotics and other pharmaceuticals, iodinated X-ray contrast media, and disinfectants that pose environmental threats, separate treatment of hospital wastewater has been considered as an alternative or in addition to municipal wastewater treatment (Ternes et al., 2004; Pauwels and Verstraete, 2006; Szekeres et al., 2017). While in the majority of cases hospital wastewater is still treated in municipal WWTPs, separate treatment systems offer opportunities to implement specialized processes for this particular type of influent (Pauwels and Verstraete, 2006; Hocquet et al., 2016). This is especially relevant in Israel, where wastewater reuse for landscape and crop irrigation is an integral part of water sustainability—over 85% of wastewater effluent is reused for irrigation and around 40% of water used in agriculture comes from treated wastewater (IWA, 2011; Reznik et al., 2017). Previous work has suggested that the influence of ARG loading from treated municipal wastewater on native soil microbiomes may not be significant (Negreanu et al., 2012; Gatica and Cytryn, 2013). However, diversity and fate of ARGs corresponding to clinically relevant antibiotics has not been thoroughly characterized in decentralized hospital wastewater treatment schemes, which impedes management of public health risks associated with onsite treatment and reuse or release of treated wastewater.

Viral communities are vastly understudied relative to their microbial counterparts in wastewater treatment infrastructure. While some research has analyzed viruses in full-scale municipal WWTPs and in biosolids (Rosario et al., 2009; Bibby and Peccia, 2013), viral diversity, community composition, and fate in on-site hospital wastewater treatment systems is not yet well understood. Research has demonstrated that wastewater contains a wide assortment of viruses from humans and other sources (Wu and Liu, 2009; Tamaki et al., 2012; Aw et al., 2014). Bacteriophages are the most common type of virus in wastewater, and human pathogens such as adenoviruses and hepatitis A viruses are also prevalent in wastewater (Prado et al., 2011; Aw et al., 2014). Reclaimed water for water reuse applications has thousands more virus-like particles per unit volume than potable water (Rosario et al., 2009). Thus, treated hospital wastewater intended for on-site irrigation reuse may also contain high viral loads compared to potable water. Furthermore, previous studies have shown conflicting results on whether or not viruses are significantly associated with ARGs and thus can facilitate their transfer between bacteria (Zhang and LeJeune, 2008; Colomer-Lluch et al., 2014; Enault et al., 2017).

Here, we focus on a unique, pilot-scale hospital wastewater treatment system located at the Tel HaShomer Hospital in Tel Aviv, Israel. This system confronts the challenge of wastewater contamination with enhanced levels of pharmaceuticals and genetic pollutants in an urban region where natural freshwater sources are limited and appropriate treatment of wastewater is imperative. The pilot-scale system treats hospital wastewater with the intent to eventually use the treated wastewater to irrigate landscaping on-site at the hospital. The objectives of this study were to investigate ARG and dsDNA viral abundances, diversity, genomic context, and fate throughout the pilot-scale hospital WWTP system, as well as to analyze corresponding bacterial community structure. We hypothesized that high levels of antibiotic-resistant bacteria in hospital wastewater facilitate persistence of multiple ARGs throughout the treatment system, yet sequential secondary treatment processes act to reduce ARG loading in effluent. We also hypothesized that the viral community could be a significant vector for ARGs in the system. This work provides important insights into the ARG and viral content of hospital wastewater and how it can potentially impact environments that receive discharged hospital effluent.

## Materials and Methods

### Pilot-Scale Hospital Wastewater Treatment System

All samples were collected from a recently constructed pilot-scale wastewater treatment system located at the Tel HaShomer Hospital in Tel Aviv, Israel. As of 2017, there were 1,389,273 patient visits in the clinics, 178,813 patient visits in the emergency room, and 1532 admission beds total. The pilot-scale system treats hospital wastewater using sequential primary and secondary treatment processes. Raw wastewater influent from the hospital is subjected to primary screening to remove large objects and particles and then is fed into a buffer tank. Primary effluent flows at an average flow rate of 40 L/h from the buffer tank into an equalization basin where pH is monitored, then into an anaerobic chamber (60 L, HRT = 1.5 h), followed by an anoxic chamber (100 L, HRT = 2.5 h), and finally an aerobic chamber (300 L, HRT = 7.5 h, DO = 2–4 mg/L) for secondary treatment. After aerobic secondary treatment, the water flows into a sedimentation tank (80 L) for biomass separation and recycling. Final effluent is currently released into sewer pipes for discharge into a municipal WWTP. The pilot-scale system is intended to mimic a system for water reuse to irrigate landscaping around the hospital, and in the future, a membrane and advanced oxidation processes will be implemented. During the time frame of sampling, chemical oxygen demand (COD) removal between influent (primary effluent) and secondary effluent averaged 57%. COD in the influent ranged from 161–307 mg/L and BOD was measured as 432 mg/L during the time frame of sampling.

### Bacterial and Viral Sampling and Concentration

Samples for shotgun metagenomics targeting the bacterial community were collected from five locations throughout the pilot-scale hospital wastewater treatment system including the influent (primary effluent) water, biomass from the anaerobic basin, biomass from the anoxic basin, biomass from the aerobic basin, and final effluent water. Influent, final effluent and biomass samples for bacterial analyses (herein termed the cellular fraction) were collected on December 5, 2018 (influent and biomass) and December 6, 2018 (final effluent) from the pilot-scale hospital wastewater treatment system. Grab samples (1 L) were taken from each water sampling location and immediately transported to the lab for processing. Water samples were then filtered through 0.22 μm pore size Sterivex^TM^ filter units (Merck Millipore, Burlington, MA, United States) and bacteria were recovered from the filters. Influent water samples were taken from the equalization basin, and 30 mL samples were filtered in duplicate. Final effluent samples (100 mL) collected after the settling tank were also filtered in duplicate. Biomass samples were collected from each of the anaerobic, anoxic, and aerobic chambers. Anaerobic and anoxic biomass samples (1.5 mL, eight replicates each) and aerobic biomass samples (50 mL, six replicates each) were centrifuged at 10,000 r/min for 8 min. Volume of samples was based on concentration of biomass in each sampling location. After centrifugation, supernatant was decanted, and biomass was stored in a −3°C freezer until DNA extraction.

Samples for viral analyses were collected from three locations throughout the system including influent water, biomass from the aerobic chamber, and effluent water. Water samples for viral analysis were collected from influent prior to the buffer tank (8 L) and final effluent (20 L) on December 9, 2018 and December 13, 2018, respectively. Sampling dates varied because viral concentration was performed on the same day as sample collection for each location and requires a full day per sample. Biomass samples for viral analysis were collected from the aerobic chamber of the pilot-scale hospital wastewater treatment system (1 L) on December 13, 2018. Influent water samples were filtered through 0.45 μm pore size PES Millipore membrane filters (MilliporeSigma, Darmstadt, Germany) prior to viral concentration to avoid clogging of dialysis filters by filtrate from these 0.45 μm pore size filters in the following step. To concentrate influent and effluent viral water samples, water was first dialyzed with Rexeed 25S Hemodialyzer filters (Asahi Kasei, Tokyo, Japan). As a result of the dialysis filtration process, 8 L of influent was concentrated to 350 mL and 20 L of effluent was concentrated to 400 mL. To lyse remaining bacterial cells, chloroform (5 mL) was added to the retentate water, vigorously mixed for 2 min, and allowed to settle for 15 min. The resulting opaque chloroform layer was then removed and the supernatant was again filtered through 0.45 μm pore size PES Millipore membrane filters and filtrate was retained for the viral samples. Samples were treated with 100 units of DNAse I (Sigma-Aldrich, St. Louis, MO, United States) per 800 μL of sample to degrade non-viral DNA based on previously described work (Kim et al., 2015).

The extraction of viruses from aerobic chamber biomass samples was performed using a protocol adapted from Guzmán et al. (2007). Briefly, biomass (45 mL) was mixed with 10% beef extract (pH 7.2) at a 1:10 ratio of biomass to beef extract and then the sample was homogenized with a Vortex at room temperature. The sample was then centrifuged for 30 min at 4000 × *g* and 4^°^C, and the supernatant was recovered. Supernatant was filtered through 0.22 μm pore size filters prior to viral DNA extraction.

### DNA Extraction

DNA from the cellular fraction was extracted from two samples per location using the FastDNA Spin Kit for Soil (MP Biomedicals, Santa Ana, CA, United States), following the manufacturer’s protocol. Genomic DNA extracts were pooled prior to DNA sequencing. Viral DNA was extracted using a QIAamp Ultrasens Virus Kit (QIAGEN, Hilden, Germany) per the manufacturer’s instructions. For viral fraction samples, DNA extracts from four biological replicates of influent were pooled, four replicates of biomass were pooled, and five replicates of effluent were pooled prior to DNA sequencing in order to obtain sufficient DNA for sequencing from each sampling location.

### High-Throughput Shotgun Metagenomics Sequencing, Processing, and Assembly

Libraries were prepared using the Nextera XT DNA kit (Illumina, San Diego, CA, United States) at the NuSeq sequencing core facility at Northwestern University. Samples were sequenced using an Illumina NextSeq500 (Illumina, San Diego, CA, United States) at NuSeq using 2 × 150 bp paired end reads. Sequences are available via NCBI BioProject accession number PRJNA526679. Sequencing and assembly statistics are summarized in Table 1. Rarefaction curves generated with non-pareil (Rodriguez-r and Konstantinidis, 2013) show sequencing coverage for each sample (Supplementary Figure S1).

**TABLE 1:** Metagenomic sequencing and assembly details.

Sample	Metagenome size (Gb)	Number of contigs	Largest contig size (bp)	N50 (bp)
				
Viral influent	8.45	91,225	257,155	1841
Viral aerobic biomass	8.09	80,533	234,988	1879
Viral effluent	5.31	80,940	289,205	1866
Bacterial anaerobic biomass	7.47			
Bacterial anoxic biomass	3.32			
Bacterial aerobic biomass	2.03			
Bacterial influent	3.04			
Bacterial effluent	5.05			
Bacterial coassembly		356,840	191,808	1984
**Total:**	42.77			
				

*Metagenome size refers to total bases in coupled reads.*

Raw reads were first screened with FastQC to assess quality, then were trimmed with CutAdapt (Martin, 2011) with a maximum allowed error rate (-e) of 0.1, quality (-q) of 20, overlap (-O) of 5, and discarded reads shorter than 20 (-m). The total size of all raw reads combined was 43.0 Gbp, and the total size of all coupled clean reads combined was 42.8 Gbp. Clean reads were then assembled into contigs using IDBA-UD (Peng et al., 2012). A minimum kmer value of 21 and a maximum kmer value of 101 were used, with a step size of 10, a minimum contig size of 500, and number of threads set to 24. Bacterial samples were coassembled, as one of the downstream goals of the study was to bin bacterial contigs into metagenome-assembled genomes (MAGs). Viral samples were assembled individually as they were not subjected to genome binning. Genome binning of bacterial samples was performed with MetaBAT (Kang et al., 2015) and genome bins were screened for quality using CheckM (Parks et al., 2015). High-quality MAG bins (> 80% completeness, < 10% contamination) were retained for downstream analyses.

### Detection of ARGs, Plasmid-Associated Genes, and Taxonomy in Bacterial Sequences

ORF detection from bacterial contigs was performed using MetaGeneMark v1 (Zhu et al., 2010). To quantify ARG abundances, predicted bacterial amino acid sequences after ORF detection were aligned with Blastp against the CARD Version 2.0.3 database (McArthur et al., 2013) using the following settings: e-value ≤ 10^–10^, percent identity ≥ 70%, and bit score ≥ 50 (Chu et al., 2018; Petrovich et al., 2018). Matching amino acid sequences were classified into ARG categories based on resistance mechanisms and the various classes of antibiotics to which the resistance genes confer resistance to. To identify plasmid-associated genes, sequences after ORF detection were aligned with Blastp against the ACLAME Version 0.4 database (Leplae et al., 2009). Coverage of genes in the cellular fraction samples was determined by mapping reads to assembled genes with Bowtie2 version 2.2.6, then gene abundances were normalized to the average of 108 single copy marker gene sequences (considered to be approximate “genome equivalents”) from the amino acid Genome Property database, entry GenProp0799—“Bacterial core gene set, exactly 1 per genome” as previously described (Chu et al., 2018; Petrovich et al., 2018). Taxonomy was assigned to high-quality bacterial genome bins using GTDB-TK based on the Genome Taxonomy Database (Parks et al., 2018). Kraken2 (Wood and Salzberg, 2014) was used to assign taxonomy to individual contigs based on the NCBI RefSeq database. These two databases were used as they pair with the tools for assigning taxonomy based on genome bins and contigs. However, taxonomy from GTDB was converted to the equivalent NCBI taxonomy using the NCBI Taxonomy Browser for class and genus-level analysis in order to be consistent.

### Bioinformatics Analysis of Viral Sequences and Statistical Analyses

To characterize taxonomic affiliation of viral sequences, assembled contigs from viral samples were aligned using Blastn against the NCBI RefSeq Viral Database (downloaded August 2018) (O’Leary et al., 2015) with e-value ≤ 10^–5^ (Tamaki et al., 2012; Kim et al., 2017) and the best alignment hits were selected for each contig. Additionally, to distinguish contigs that may contain any viral sequences from contigs predicted to be primarily viral, Blastn results were further parsed to identify contigs where the alignment length matched the reference over at least 80% of its length with over 95% identity. These contigs were inferred to be primarily viral and were compared to contigs containing any viral sequences from Blast results. Sequences from viral samples were screened to determine the degree of cellular genomic DNA contamination using VirSorter software (Roux et al., 2015). Taxonomic identification of viruses was based on lineage information from NCBI. Relative abundance of individual viral families was normalized to total coverage of all identified viruses in each sample. Putative bacterial hosts to viruses were predicted using the GenomeNet Virus-Host Database, based on the sequence similarity of viral sequences in contigs here to known viruses with known hosts associations (Mihara et al., 2016). Coverages of predicted hosts of viruses in viral fraction metagenomes from the Virus-Host Database at the class and genus level were divided by total coverage of all viruses in a given sample to show what proportion of total viruses each class or genus comprised. Beta diversity analyses and corresponding PERMANOVA statistics of abundances of predicted bacterial hosts to viruses, bacteria harboring ARGs, and bacteria from the overall bacterial community were calculated with the Scikit-Bio Python package^1^ using a Bray-Curtis distance matrix. Principal coordinates analysis graphs representing this beta diversity were created with the Matplotlib^2^ and Seaborn^3^ Python packages.

## Results

### Abundance, Diversity, and Mobilization of ARGs in Bacterial Samples

**FIGURE 1:**
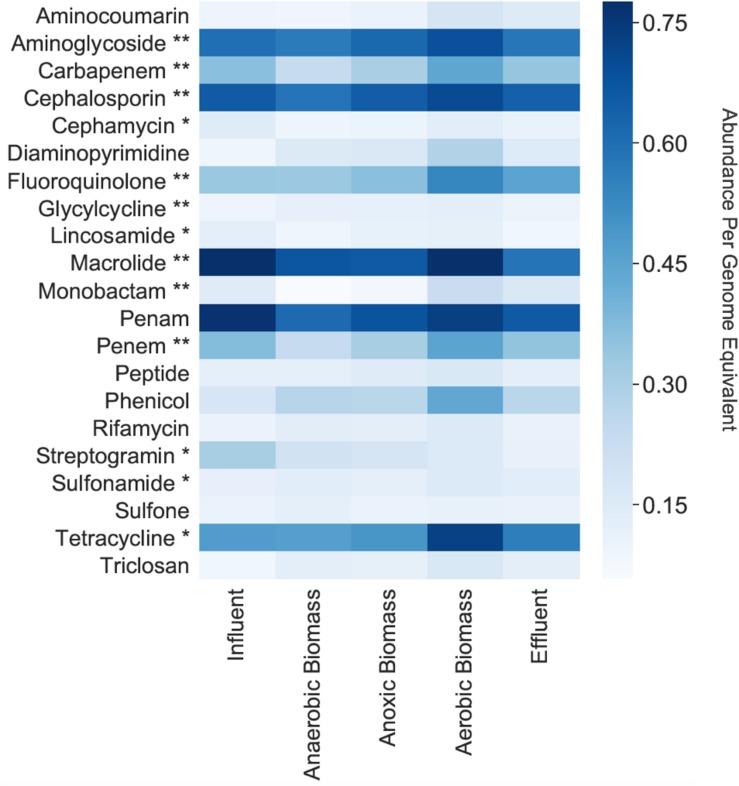
Normalized relative abundances per genome equivalent of antibiotic resistance gene categories in the cellular fraction (samples collected for bacterial analyses). **Critically important according to the WHO (Collignon et al., 2016). *Highly important according to the WHO (Collignon et al., 2016).

Among the cellular fraction metagenomes from the influent, anaerobic biomass, anoxic biomass, aerobic biomass, and effluent samples of the hospital wastewater treatment system, we identified 264 unique ARGs conferring resistance to 22 classes of antibacterial drugs. The most common categories of ARGs detected throughout the system are shown in Figure 1. The number of unique ARGs detected in each sampling location varied little across the system, ranging from 224 in anaerobic biomass to 248 in effluent. Samples contained unique ARGs such that intermediate sampling locations throughout the system contained ARGs that were not necessarily present in the effluent. The ARGs included those conferring resistance to antibiotics that the World Health Organization (WHO) has highlighted as “critically important” and “highly important” for human medicine (Collignon et al., 2016). We found ARGs conferring resistance to aminoglycoside, cephalosporin, macrolide, penam, tetracycline, and fluoroquinolone antibiotics throughout the treatment system at relative abundances of 0.60, 0.66, 0.78, 0.77, 0.47, and 0.33 copies per genome equivalent, respectively, in influent, and at relative abundances of 0.58, 0.64, 0.59, 0.66, 0.56, and 0.45 copies per genome equivalent, respectively, in effluent. Aminoglycoside, cephalosporin, fluoroquinolone, and macrolides are all considered to be critically important antibiotic classes, and tetracycline is highly important. Other classes of antibiotics considered critically important by the WHO, including carbapenem, glycylcycline, and monobactam, and highly important, including cephamycin, lincosamide, streptogramin, and sulfonamide, were also represented in the system but in lower relative abundances (Figure 1).

**FIGURE 2:**
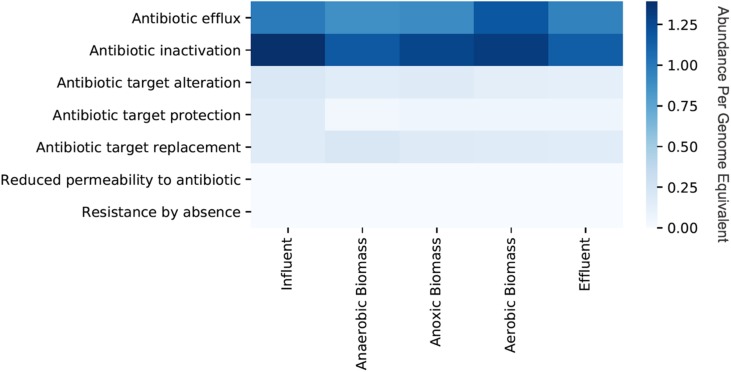
Relative abundances per genome equivalent of antibiotic resistance genes categorized by resistance mechanism from the cellular metagenomes. “Cellular metagenomes” refers to metagenomes from samples collected for bacterial analyses.

Persistence and fate of ARGs in the cellular fraction samples throughout the transect of the wastewater treatment system varied for different classes of ARGs (Figure 1). Only a slight decrease of total ARGs (16% overall ARG removal per genome equivalent) was observed over the course of treatment between influent and effluent. ARGs were slightly more abundant in influent than effluent for some classes on a per genome equivalent basis such as macrolide (0.78 in influent and 0.59 in effluent), penam (0.77 in influent and 0.66 in effluent), and streptogramin (0.31 in influent and 0.11 in effluent). By contrast, some classes of ARGs increased in abundance per genome equivalent between influent and effluent such as those conferring resistance to fluoroquinolone (0.33 in influent and 0.45 in effluent) and tetracycline (0.47 in influent and 0.56 in effluent). Aerobic biomass had more ARGs overall compared to both anaerobic and anoxic biomass. All of the identified ARGs corresponded to seven different resistance mechanisms, with antibiotic efflux and antibiotic inactivation most common in all samples, although the relative abundances differed between sampling locations in the treatment system (Figure 2). Antibiotic efflux ARGs had similar relative abundances between influent and effluent (1.0 copies/genome equivalent), and were most abundant in aerobic biomass (1.2 copies/genome equivalent). Antibiotic inactivation ARGs were highest in influent (1.4 copies/genome equivalent) and less abundant in the effluent (1.1 copies/genome equivalent).

**FIGURE 3:**
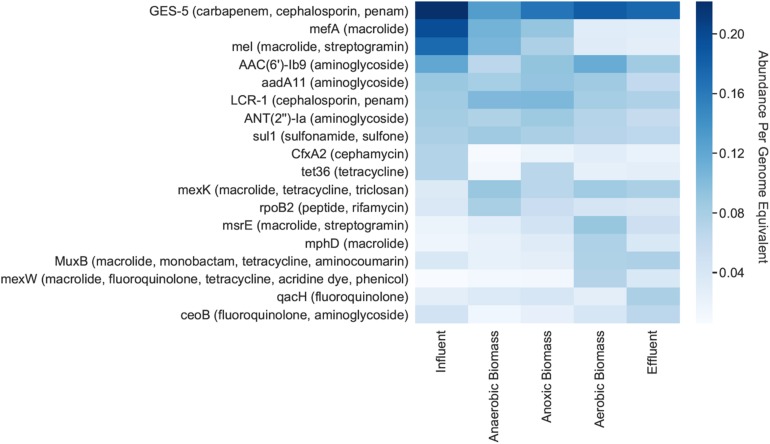
Relative abundances per genome equivalent of individual antibiotic resistance genes. The 10 most abundant ARGs for each cellular metagenome are shown, along with the antibiotic(s) that each gene putatively confers resistance to (in parentheses). “Cellular metagenomes” refers to metagenomes derived from samples collected for bacterial analyses.

Individual ARGs were identified on bacterial contigs throughout the pilot-scale system (Figure 3). GES-5, which confers resistance to carbapenem, cephalosporin, and penam antibiotics, had the highest relative abundance per genome equivalent throughout the system, including in the influent where there were 0.22 copies/genome equivalent (Figure 3). Other genes exhibiting high abundance in the influent included *mefA* (macrolide resistance), *mel* (macrolide and streptogramin resistance), and *AAC(6’)-Ib9* (aminoglycoside resistance) which had relative abundances of 0.20, 0.17, and 0.12 copies/genome equivalent, respectively. The *mefA* and *mel* genes both decreased in effluent to 0.03 copies/genome equivalent for each gene.

Additionally, we looked for the co-occurrence of ARGs and plasmid-associated genes that can promote their mobilization between bacteria. The percentages of ARGs associated with plasmid-associated genes were 42, 49, 51, 48, and 52% in influent, effluent, anaerobic biomass, anoxic biomass, and effluent samples, respectively, indicating a high proportion of ARGs likely associated with horizontal gene transfer in all samples and a possible but small enrichment in the effluent compared to in the influent.

### Metagenome-Assembled Genomes

Contigs from bacterial samples were assembled into MAGs in order to gain a more thorough understanding of genetic content of individual taxa, evaluate the genomic context of antibiotic resistance determinants, and identify high abundance taxa harboring genomic capacity for multidrug resistance. A total of 19 high-quality (> 80% completeness, < 10% contamination) MAGs were assembled, representing 2.2, 1.9, 2.3, 3.1, and 4.5% of the total communities in influent, anaerobic biomass, anoxic biomass, aerobic biomass, and effluent samples, respectively, based on contig coverage (Supplementary Table S1). Of the 19 MAGs, five contained a single ARG. These genomes were phylogenetically affiliated with *Bifidobacterium adolescentis B*, *Methylobacillus*, *Dechloromonas*, *Brachymonas*, and *Bifidobacterium longum*. Another five MAGs contained multiple ARGs, including some that confer resistance to multiple antibiotics, and were phylogenetically affiliated with *Acinetobacter towneri*, *Tolumonas*, *Gemmobacter*, *Burkholderiaceae*, and *Tolumonas auensis*. For example, genome bin #18 (Supplementary Table S1) identified as *T. auensis* contained the *CRP* gene which is associated with resistance to fluoroquinolone, penam, and macrolide antibiotics, *ugd* which confers resistance to peptide antibiotics, and *mdtF* which is involved with efflux of penam, macrolide, and fluoroquinolone antibiotics.

### Composition and Diversity of Viral Populations

Caudovirales was the dominant order of viruses, making up 72%, 71%, and 88% of sequences identified as viral based on contig coverage in the influent, biomass, and effluent, respectively. *Myoviridae*, *Podoviridae*, and *Siphoviridae* were the most common families of viruses, together accounting for 71.9% of identifiable viruses in influent, 70.6% in biomass, and 88.0% in effluent (Supplementary Figure S2). All three of these families belong to the order Caudovirales, which are tailed bacteriophages. *Myoviridae*, *Podoviridae*, and *Siphoviridae* were also the most common families identified when only dominantly viral contigs (> 80% of reference length, > 95% identity) were included in the taxonomic analysis (Supplementary Figure S3).

Less than 3% of viral sequences (normalized to contig coverage) in any sample could be taxonomically assigned based on the NCBI Viral RefSeq database. When samples were screened for viral content with VirSorter (Roux et al., 2015), fewer than 2% of contigs (normalized to contig coverage) were assigned as exclusively viral and non-prophage. Bacterial contamination in metagenomic samples prepared using viral concentration and purification methods is common (Roux et al., 2013), but some of these sequences could be identified as prophages by VirSorter. These prophages accounted for 0.11, 0.19, and 0.03% relative abundance in the viral influent, biomass, and effluent samples, respectively.

Contigs from the viral metagenomes were screened for bacterial ARGs in order to identify potential for horizontal transfer of these genes. Out of all contigs in viral samples that contained taxonomically identifiable viral sequences by the RefSeq database, only a single contig with a positive viral hit in the NCBI RefSeq Viral Database also contained an ARG based on alignment against the CARD database. This contig was found in the effluent and contained viral DNA from the family *Myoviridae* as well as the ARG *VCC-1*, which confers resistance to monobactam, carbapenem, and penam antibiotics. However, this contig only contained 2.4% viral DNA (99 bp aligned as viral out of a total contig length of 4184 bp), and thus was not in our contig group of likely viral sequences. Therefore, no strong linkages were observed between viral sequences and ARGs in the viral metagenomes. From the coassembled cellular fraction metagenomes, seven contigs were identified that contained some ARG DNA and viral DNA, but relative abundances of these contigs were very low in the system based on coverage (0.0007, 0.0001, 0.0002, 0.0003, and 0.0003% of total contigs on average for influent, anaerobic biomass, anoxic biomass, aerobic biomass, and effluent, respectively).

### Taxonomic Composition of Total Bacteria, Bacteria Harboring ARGs, and Predicted Bacterial Hosts of Viruses

**FIGURE 4:**
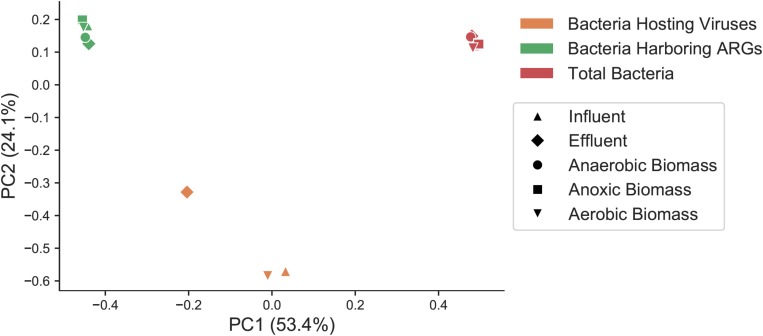
Principal coordinates analysis (PCoA) representing sample beta diversity for all bacterial communities, the subset of bacteria harboring ARGs, and the subset of predicted bacterial hosts of viruses. Distances are based on the Bray–Curtis dissimilarity measures at the genus level.

In order to determine if the same types of bacteria harbored ARGs and viruses, and to assess how bacteria harboring ARGs and bacteria predicted to be the host for phages compared to the overall bacterial community, we categorized bacterial taxa at the class level in the overall bacterial community (Supplementary Figure S4A), the subset predicted to harbor ARGs (Supplementary Figure S4B), and the predicted bacterial hosts for viral sequences (Supplementary Figures S3, S4C) in each sample throughout the treatment plant. Total bacteria, the subset of those bacteria that harbored ARGs, and the predicted bacterial hosts to viruses were all significantly distinct communities from one another. Beta diversity of total bacteria, the fraction of bacteria harboring ARGs, and predicted bacterial hosts to viruses was compared at the genus level with a Bray–Curtis distance matrix, as many predicted viral hosts were not assigned at the species level (Figure 4). These three groups showed statistically significant differences from one another (PERMANOVA pseudo-F test statistic = 15.8, *p* < 0.01).

For contigs that were assigned to high-quality MAGs, taxonomy was based alignments of binned contigs in the entire MAG against the Genome Taxonomy Database, and then that taxonomy was converted to equivalent NCBI taxonomy for consistency. For contigs that were not assigned to high-quality MAGs, taxonomy was assigned with Kraken2 which also uses NCBI taxonomy. Betaproteobacteria was the dominant class of bacteria overall in all samples (30–49%), and Gammaproteobacteria were the second most dominant class (14–27%). Betaproteobacteria were more abundant in effluent (49%) compared to influent (34%). Gammaproteobacteria abundance varied over the course of treatment, comprising 14% of influent and 17% of effluent, and was highest in aerobic biomass at 27%. Among both bacteria carrying ARGs and populations of bacteria predicted to be the hosts for phages, Gammaproteobacteria was by far the most dominant class in all samples, making up 44–61% of bacteria carrying ARGs and 38–45% of predicted viral hosts. Bacilli and Betaproteobacteria were the other two most represented classes of bacteria carrying ARGs, but those classes made up only 3–14 and 9–19%, respectively, of ARG-harboring bacteria.

## Discussion

### Public Health Risks Associated With Hospital Wastewater

Hospital wastewater contains a complex mixture of pharmaceuticals, ARGs, pathogenic microorganisms, and viruses that can threaten human health, and as a result, this type of wastewater is particularly concerning for public health protection (Gautam et al., 2007; Duong et al., 2008; Chagas et al., 2011; Fuentefria et al., 2011; Prado et al., 2011; Korzeniewska et al., 2013; Rodriguez-Mozaz et al., 2015; Hocquet et al., 2016). In most countries, hospital wastewater is discharged into municipal sewage systems without any required pretreatment, and the European Directive 91/271/EEC does not place restrictions on discharge of this type of wastewater (Hocquet et al., 2016; Szekeres et al., 2017). The pharmaceutical compounds in hospital wastewater can require targeted and advanced removal strategies beyond what is performed by municipal WWTPs, and the presence of antibiotics can create selective pressures for antibiotic resistance in bacteria (Martinez, 2009; Gao et al., 2012). Thus, the pilot-scale system in this study is innovative as it aims to treat hospital wastewater separately from municipal wastewater to remove pathogenic bacteria and harmful chemicals. The pilot-scale system currently discharges its effluent into the municipal sewer system, but mimics a system for water reuse and eventually plans to reuse treated effluent for on-site landscape irrigation once additional tertiary treatment components are implemented.

Here, we found that both the influent and effluent from a hospital wastewater treatment system contained ARGs conferring resistance to a variety of antibiotics, including classes with high clinical importance, as well as individual ARGs such as *sul1*, *mefA*, and *mel* that are considered to be clinically concerning. Some of these genes can be commonly found in environments other than hospital wastewater (Zhou et al., 2017), yet bacteria that harbor clinically relevant ARGs are particularly concerning in hospital settings where infections be can resistant to treatment by commonly used antibiotics. In Brazil, multidrug resistant *Pseudomonas aeruginosa* was detected in wastewater from two different hospitals, and this bacterium demonstrated simultaneous resistance to up to nine different antibiotics that were tested using culture-based techniques (Fuentefria et al., 2011). Other research has shown that hospital wastewater contains higher fractions of antibiotic-resistant *P. aeruginosa* and vancomycin-resistant enterococci than municipal wastewater (Hocquet et al., 2016). *Escherichia coli* isolates from hospital wastewater in Poland exhibited higher overall resistance to antibiotics than isolates from municipal wastewater, and up to 37% of hospital wastewater isolates contained extended-spectrum beta-lactamases (ESBLs) compared to 18% in municipal wastewater (Korzeniewska et al., 2013). In the present study, use of a genome-resolved shotgun metagenomics approach provides a broad and comprehensive view of ARG abundance, diversity, and genomic context, and provides insight into ARG composition in hospital wastewater treated on-site. In particular, we found many ARGs with high relative abundances were associated with resistance to a variety of antibiotics (Figure 3), and identified organisms that might harbor multiple ARGs (Supplementary Table S1).

### Antibiotic Resistance in the Pilot-Scale Hospital Wastewater Treatment System

Some abundant ARGs that we detected in hospital influent and effluent, such as *sul1*, were found to dominate hospital wastewater from other regions (Rodriguez-Mozaz et al., 2015; Szekeres et al., 2017). Other ARGs that we identified in this system such as *bla*_*TEM*_, *bla*_*OXA*_, *ermB*, *tetO*, *tetW*, and *mefA* have also been found in hospital wastewater from around the world (Chagas et al., 2011; Rodriguez-Mozaz et al., 2015; Varela et al., 2016; Ng et al., 2017; Szekeres et al., 2017). Three of the ARGs that we found in this treatment system, *mefA* and *mel*, both of which confer resistance to macrolides, and *GES-5*, which confers resistance to both carbapenems and cephalosporins, are considered to be critically important by the WHO. According to the WHO, critically important classes of antibiotics for treating humans fit two criteria: (1) the antibiotic class is either the only or one of very few options for treating severe infections and (2) it can treat infections due to bacteria that humans can acquire from non-human sources or due to bacteria that can obtain genes conferring resistance from non-human sources. Highly important classes of antibiotics only fit one of these criteria (Collignon et al., 2016).

Among clinically relevant ARGs, *mcr-1*, which encodes resistance to the last-resort antibiotic polymyxin-E (Hembach et al., 2017), the vancomycin resistance gene *vanA*, and the beta-lactam resistance gene CTX-M-32 (Volkmann et al., 2004; Hembach et al., 2017) were not detected in any sample from this system. However, a variety of clinically relevant ARGs such as the aminoglycoside ARG *aadA*, the beta lactam resistance genes *bla*_TEM–__1_, *bla*_NDM–__1_, *bla*_OXA–__2_, *bla*_OXA__35_, and *bla*_OXA–__10_, the macrolide ARGs *mefA* and *mel*, the tetracycline ARG *tetX*, and the sulfonamide ARG *sul1* (Szczepanowski et al., 2009; Devarajan et al., 2015; Hembach et al., 2017; Ng et al., 2017) were detected in most samples. The genes *mefA*, *tetX*, *bla*_OXA–__10_, and *sul1* occurred at ≥ 0.06 copies per genome equivalent in influent and all three biomass samples, whereas the other clinically relevant ARGs listed above showed < 0.06 copies/genome equivalent at all sampling points.

The diverse composition of ARGs and presence of clinically relevant genes in each sampling location support the original hypothesis that hospital wastewater facilitates presence of antibiotic resistance throughout the pilot-scale system. However, the sequential secondary treatment processes in this system did not reduce ARG loading to the extent that has been previously observed in municipal WWTPs (Petrovich et al., 2018). ARGs per genome equivalent were 16% lower overall in the effluent than in the influent in the cellular fraction, a proxy for overall removal efficiency. This is significantly lower than in our previous work, which showed that ARGs in two full-scale municipal WWTPs decreased by more than 90% on a per genome equivalent basis between influent and effluent (Petrovich et al., 2018). Indeed, while most ARG classes decreased somewhat in abundance throughout the treatment process, a few (namely, fluoroquinolone and tetracycline ARGs) were slightly higher on a genome equivalent basis in effluent than in influent. While the reasons for this are not known, this observation supports the contention that the secondary treatment process was not effective at limiting ARG loading. It should be noted that only single time points were used in each of these studies, so the conclusions must be considered in that context. It is possible that decreases in average ARGs per cell in the municipal WWTPs as well as in the pilot-scale system could be explained by the fact that anaerobic enteric bacteria in influent do not typically survive well in WWTP environments (Wells et al., 2014). Aminoglycoside ARGs were present at similar abundances (∼0.6–0.7 copies per genome equivalent) at all sampling locations throughout the hospital WWTP, and aminoglycoside ARGs were also the most consistently abundant at all sampling points throughout both of these municipal WWTPs. However, other classes of ARGs such as those conferring resistance to cephalosporins, fluoroquinolones, macrolides, penams, and tetracyclines were found in effluent at similar abundances to the influent (with at least 0.4 copies per genome equivalent in effluent), whereas all classes of ARGs in the municipal WWTPs other than those conferring resistance to aminoglycoside and beta-lactam antibiotics were reduced to abundances below 0.1 copies per genome equivalent (Petrovich et al., 2018). In contrast with full-scale municipal WWTP systems, the pilot-scale system is relatively small and treats hospital wastewater, so these differences in scale, stability, and influent composition could influence the fate of ARGs throughout the treatment processes.

### Associations Between ARGs, Viruses, and Bacteria

There has been some debate in the literature as to whether viruses are associated with ARGs and therefore play a role in their horizontal gene transfer (Zhang and LeJeune, 2008; Colomer-Lluch et al., 2014; Enault et al., 2017). We were not able to establish a strong link between viral sequences from viral metagenomes and ARGs, indicating that viruses may not be a quantitatively important vector for ARG transfer via transduction in this system. An analysis of viral metagenomes from human- and mouse-associated environments by Enault et al. (2017) found that ARGs were infrequently associated with phages, which aligns with our observations. The authors point out that viral metagenomes often contain traces of contaminant bacterial DNA and false positives are possible (Roux et al., 2013; Enault et al., 2017). Other research has suggested that viruses can indeed facilitate transfer of ARGs between bacteria. A study by Zhang and LeJeune (2008), for example, showed that tetracycline and beta-lactam resistance could be transferred by phage transduction between two different species of *Salmonella*. Transduction of ARGs has also been demonstrated in phages infecting *S. aureus* (Varga et al., 2012; Haaber et al., 2016). Recently, it has also been demonstrated for genes conferring resistance to arsenic (Tang et al., 2019). Transduction is common in WWTPs as a result of high bacterial and viral density, and it is estimated that each hour thousands of transductions occur in these environments (Rizzo et al., 2013; Lood et al., 2017). Therefore, in certain environments, it is possible that viruses may be associated with ARG transfer (Fernández-Orth et al., 2019). In addition, recent work has suggested phage-inducible chromosomal islands are widespread amongst bacteria, facilitating transduction of ARGs and virulence factors (Chen et al., 2015; Novick and Ram, 2016; Martínez-Rubio et al., 2017; Fillol-Salom et al., 2018). We adopted a conservative approach for identifying ARGs on contigs containing viral DNA in this study, and therefore only evaluated the potential for transducing particles containing viral and host DNA on the same strand of DNA neglecting ARGs associated with phage-inducible chromosomal islands. Future work is needed to characterize transducing particles with bacterial DNA on separate DNA strands than viral DNA to act as vectors for ARGs.

Overall, bacterial community composition included similar class representation to findings from previous studies on hospital wastewater (Szekeres et al., 2017) and municipal wastewater (Bengtsson-Palme et al., 2016), including Gammaproteobacteria making up a significant portion of the total population. Betaproteobacteria made up around 20–25% of total bacteria in a Swedish municipal wastewater system (Bengtsson-Palme et al., 2016) and less than 10% of total bacteria in wastewater from a hospital in Romania (Szekeres et al., 2017), lower than its relative abundance of 30–49% here.

The community composition of viruses that we identified agrees with other findings which have shown that the families *Myoviridae*, *Podoviridae*, and *Siphoviridae* from the order Caudovirales representing tailed bacteriophages made up the vast majority of detected viral communities in wastewater (Rosario et al., 2009; Parsley et al., 2010; Cantalupo et al., 2011; Tamaki et al., 2012). Phage outnumber human pathogens in wastewater, and while it is possible that some human viral pathogens may have been present, they were not captured by the given sequencing depth. In terms of bacterial hosts, the finding that Gammaproteobacteria were the most common predicted viral hosts in wastewater (38–44% of total predicted hosts) aligns reasonably well with a previous metagenomics analysis of hospital wastewater which investigated contigs containing both bacterial and phage annotations (Subirats et al., 2016). That study found that virus families *Myoviridae*, *Podoviridae*, and *Siphoviridae* were commonly associated with Gammaproteobacteria, and to a lesser extent with Alphaproteobacteria, Betaproteobacteria, and Firmicutes (Subirats et al., 2016). By contrast, we found that Betaproteobacteria was a significant putative viral host class while Alphaproteobacteria and Firmicutes made up much smaller fractions of predicted viral hosts in the pilot-scale system.

Our viral survey was limited by the potential drawbacks inherent in viral metagenomics: the majority of viral sequences in metagenomes are not assigned due to the high number of unclassified viruses and resulting database limitations, indicating a high degree of viral diversity beyond what can be currently described by taxonomic annotation (Cantalupo et al., 2011; Tamaki et al., 2012). Our study only targeted dsDNA viral sequences, which further limits the scope of diversity that can be characterized. Viral reads can be difficult to assemble, as viral genomes contain repeat regions and exhibit substantial intrapopulation diversity and variation (Smits et al., 2014; Rose et al., 2016; Sutton et al., 2019). Uneven coverage of sequences can pose an additional assembly challenge (Smits et al., 2014; Rose et al., 2016). Thus, while shotgun metagenomics is a useful tool for studying these communities, results must be placed in the context of the challenges associated with this approach. In addition, it should be noted that bacterial and viral grab samples were collected on two different dates. Future work is warranted to evaluate temporal changes in viral and bacterial communities, and associations with ARGs, to identify how these change with time.

## Conclusion

The results from this study reveal that ARGs, including many that confer resistance to antibiotics of high clinical relevance, were abundant and persistent throughout a pilot-scale hospital wastewater treatment system in Tel Aviv, Israel. Individual ARGs of significant clinical relevance including *aadA*, *bla*_TEM–__1_, *bla*_NDM–__1_, *bla*_OXA–__2_, *bla*_OXA__35_, *bla*_OXA–__10_, *mefA, mel*, *tetX*, and *sul1* were found in most samples from throughout the hospital WWTP. This wastewater treatment system is unique as it treats hospital wastewater on-site with the aim to eventually reuse effluent for on-site landscape irrigation, in contrast to many hospitals worldwide that discharge untreated wastewater to sewer systems for treatment in full-scale municipal WWTPs. The overall removal efficiency of ARGs throughout the pilot-scale system was only 16% on a per genome equivalent basis. These findings are important because they highlight potential threats to public health through dissemination of ARGs via wastewater effluent, particularly in regions such as Israel where wastewater reuse for irrigation is very common. When we evaluated bacterial community structure in the pilot-scale system, we found that the overall bacterial community, the subset of bacteria that harbored ARGs, and predicted bacterial hosts of viruses were all significantly distinct from one another. Therefore, certain types of bacteria tend to harbor ARGs, or are likely to host viruses, and these unique subsets of the overall bacterial community that harbor ARGs and that host viruses are unique from the total community profiles. This aligns with the fact that we did not identify strong linkages between ARGs and viruses in viral metagenomes, which suggests that the viral community may not play a significant role in promoting horizontal gene transfer of ARGs in this hospital wastewater treatment system. It should be noted, however, that there is still much to be learned about the propensity of viruses to transfer ARGs between different bacteria, and conflicting results from previous studies warrant further research on this topic. Further, we note that the virus-host predictions are possible only for viruses and viral contigs similar to known viruses (O’Leary et al., 2015), which is not likely to represent the majority of viruses in our datasets. The large number of unassigned viral sequences in this study and others also highlights a need for deeper exploration into unknown viral species coupled with annotation of viral taxa in sequence databases to allow for more thorough future analyses of viral communities in metagenomes, including those representing hospital wastewater.

## Data Availability Statement

The datasets generated in this study can be found in the NCBI repository (https://www.ncbi.nlm.nih.gov/bioproject) under the BioProject ID PRJNA526679.

## Author Contributions

MP collected and processed samples from the hospital wastewater treatment system, extracted DNA, analyzed metagenomics sequence data, created figures, and wrote the main content of the manuscript. AZ managed operation of the pilot-scale system and assisted with sample collection. AK and GE assisted with sample collection and processing. YW assisted with taxonomic annotation of genome bins. KL, MD, and KW developed methods for viral concentration and extraction and contributed to development of the manuscript. RP helped plan the project and contributed to development of the manuscript. DA hosted MP and GW at the Water Research Center at Tel Aviv University, was involved with project planning and grant preparation, and assisted with sample collection. GW participated in project planning, grant proposal writing, sample collection, DNA extraction, and manuscript development.

## Conflict of Interest

The authors declare that the research was conducted in the absence of any commercial or financial relationships that could be construed as a potential conflict of interest.

## Footnotes

1 http://scikit-bio.org/

2 https://matplotlib.org/

3 https://seaborn.pydata.org/
