# Supplementary material for: Microbial and Viral Communities and Their Antibiotic Resistance Genes Throughout a Hospital Wastewater Treatment System
Source: Front Microbiol. 2020 Feb 19;11:153. doi: 10.3389/fmicb.2020.00153 (PMC7042388; doi:10.3389/fmicb.2020.00153)
Supplement: Supplementary file 1 [file Data_Sheet_1.DOCX]

**Supplementary Material**


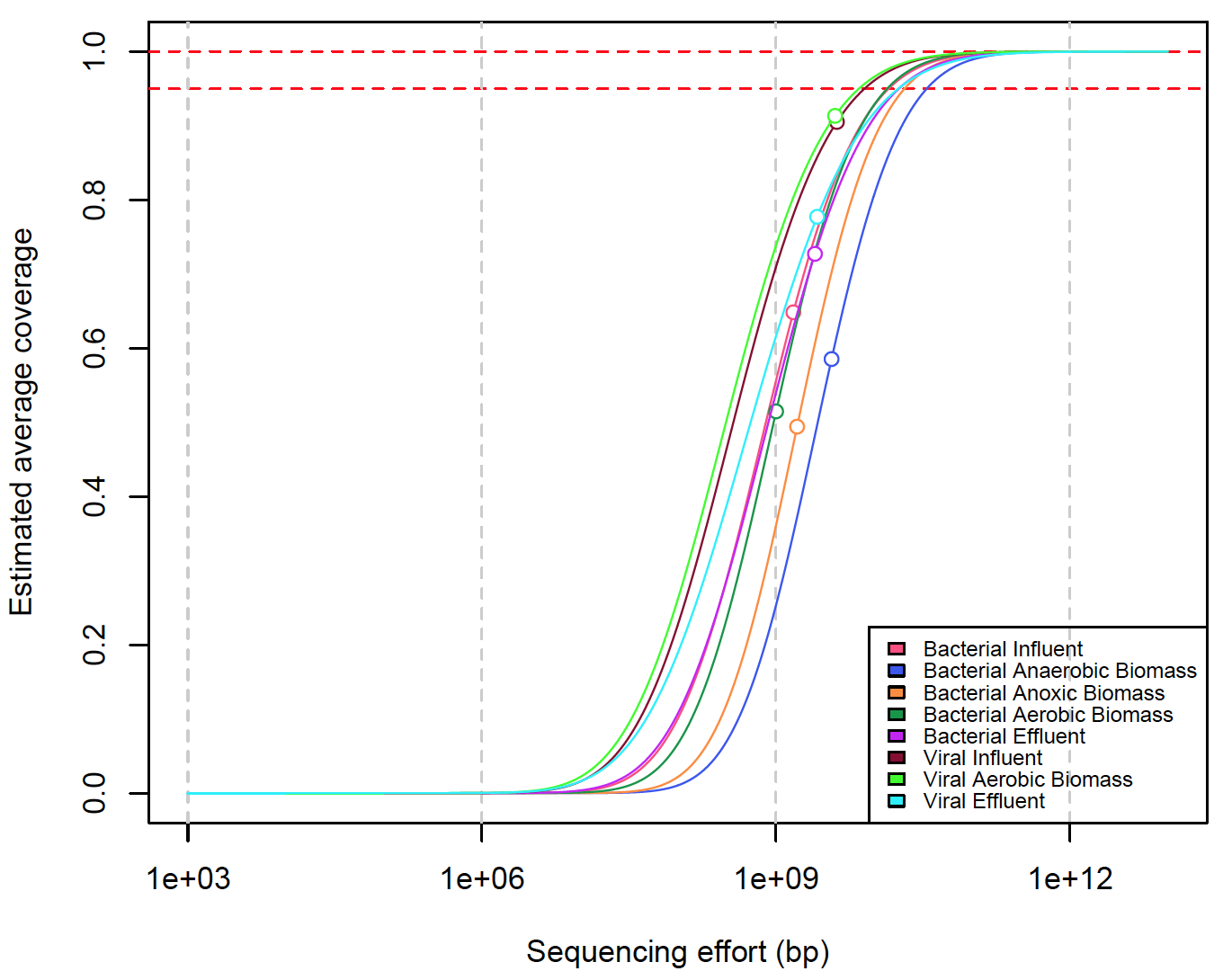


**Figure S1:** Estimated coverage of metagenomic datasets for all bacterial (cellular fraction) and viral samples. Dashed red lines represent 95-100% estimated average coverage. Nonpareil curves were constructed using the protocol in Rodriguez & Konstantinidis 2013.

**Figure S2:** Taxonomy of dsDNA viruses from all viral sequences detected with e-value ≤ 10^-5^ in the HWW treatment system at the family level. Relative abundance refers to percentage of total viral coverage in each sampling location.

**Figure S3:** Taxonomy at the family level of contigs that aligned with the NCBI RefSeq Viral Database for >80% of contig length at >95% identity in viral samples from the HWW treatment system.

(A)


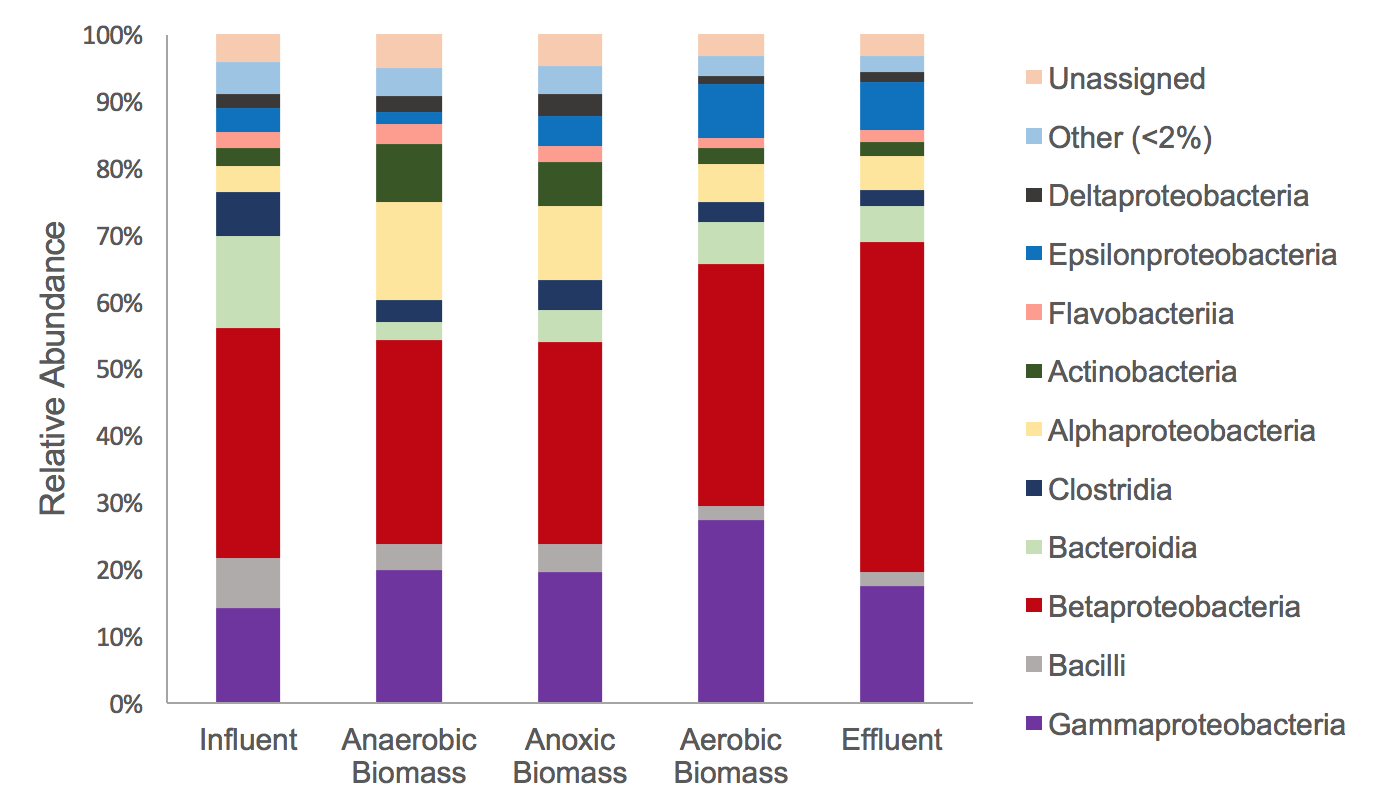


(B)


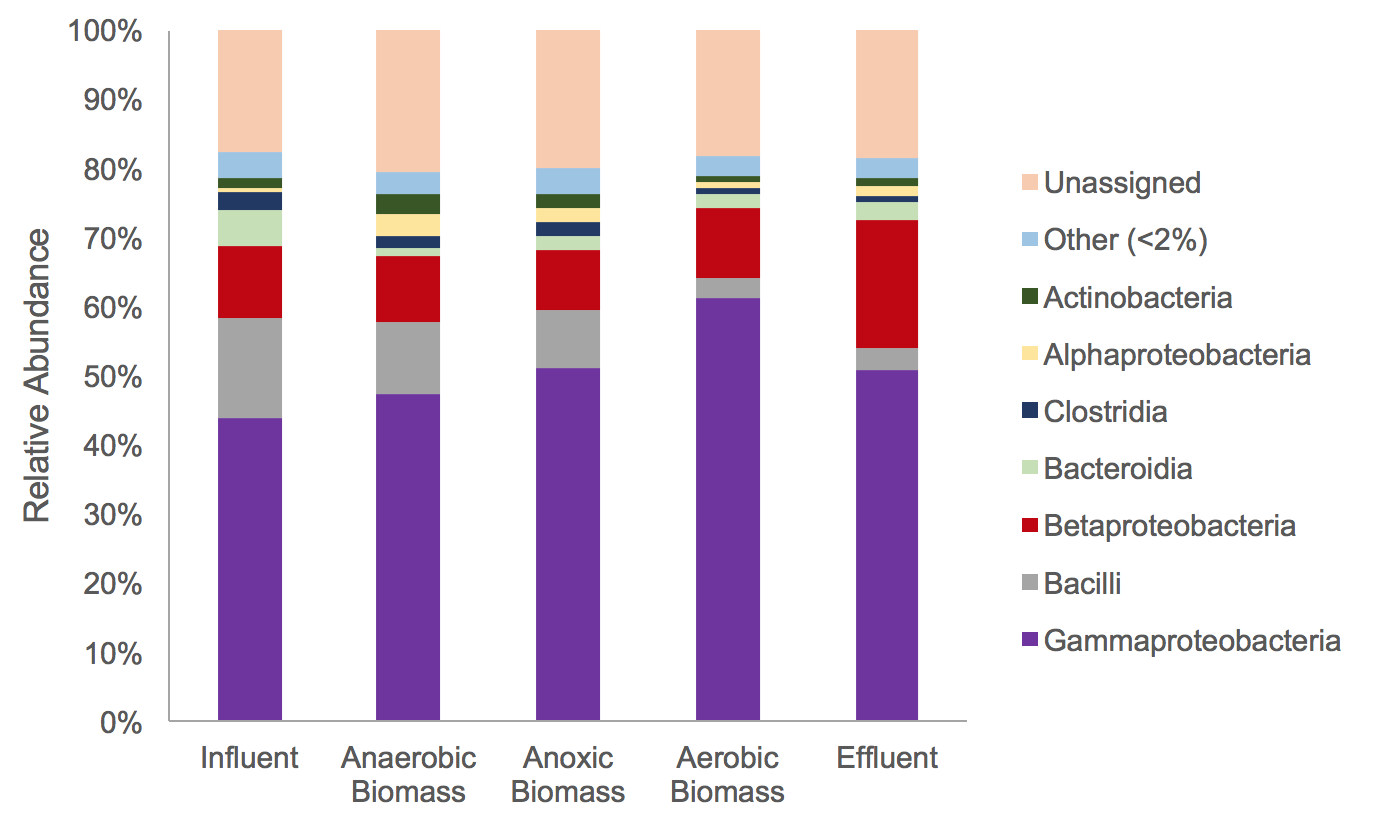


(C)

**Figure S4:** Taxonomic composition of bacteria at the class level based on NCBI classification. Relative abundance refers to percentage of total coverage. **A)** Overall bacterial community. **B)** Bacteria associated with ARGs. **C)** Predicted bacterial hosts for viruses from all viral sequences detected with e-value ≤ 10^-5^.

**Table S1:** Characteristics of high quality (>80% completeness, <10% contamination) metagenome assembled genomes from the cellular fraction (samples collected for bacterial analyses), including taxonomy and ARG content.

| **Genome Bin** | **Taxonomy** | **Number of Scaffolds** | **Completeness (%)** | **Contamination (%)** | **Genome Size (Gbp)** | **N50 (bp)** | **ARGs** |
| --- | --- | --- | --- | --- | --- | --- | --- |
| 1 | **GTDB:** Bacteria; Actinobacteriota; Actinobacteria; Actinomycetales; Bifidobacteriaceae; *Bifidobacterium; Bifidobacterium adolescentis B*  **NCBI:** Bacteria; Actinobacteria; Actinobacteria; Bifidobacteriales; Bifidobacteriaceae; *Bifidobacterium; Bifidobacterium adolescentis* | 205 | 90 | 7 | 1.83 | 11,817 | *ileS* |
| 2 | **GTDB:** Bacteria; Proteobacteria; Gammaproteobacteria; Betaproteobacteriales; Methylophilaceae; *Methylobacillus*  **NCBI:** Bacteria; Proteobacteria; Betaproteobacteria; Nitrosomonadales; Methylophilaceae; *Methylobacillus* | 112 | 96 | 0 | 2.26 | 31,062 | *amrB* |
| 3 | **GTDB:** Bacteria; Firmicutes; Clostridia; Christensenellales; CAG-138; *UBA7703*  **NCBI:** Bacteria; Firmicutes; Clostridia | 151 | 96 | 2 | 2.60 | 30,938 |  |
| 4 | **GTDB:** Bacteria; Campylobacterota; Campylobacteria; Campylobacterales; Sulfurospirillaceae; *Sulfurospirillum; GCF_001548035.1*  **NCBI:** Bacteria; Proteobacteria; Epsilonproteobacteria; Campylobacterales; Campylobacteraceae; *Sulfurospirillum* | 174 | 99 | 2 | 2.46 | 23,639 |  |
| 5 | **GTDB:** Bacteria; Proteobacteria; Gammaproteobacteria; Betaproteobacteriales; Rhodocyclaceae; *Dechloromonas*  **NCBI:** Bacteria; Proteobacteria; Betaproteobacteria; Rhodocyclales; Azonexaceae; *Dechloromonas* | 403 | 83 | 5 | 2.82 | 8,523 |  |
| 6 | **GTDB:** Bacteria; Firmicutes; Clostridia; Clostridiales; Clostridiaceae; *Proteiniclasticum; UBA8860*  **NCBI:** Bacteria; Firmicutes; Clostridia; Clostridiales; Clostridiaceae; *Proteiniclasticum* | 261 | 84 | 4 | 2.68 | 14,846 |  |
| 7 | **GTDB:** Bacteria; Proteobacteria; Gammaproteobacteria; Pseudomonadales; Moraxellaceae; *Acinetobacter; Acinetobacter towneri*  **NCBI:** Bacteria; Proteobacteria; Gammaproteobacteria; Pseudomonadales; Moraxellaceae; *Acinetobacter; Acinetobacter towneri* | 333 | 86 | 2 | 2.30 | 8,547 | *adeK, adeJ, adeI, abeM* |
| 8 | **GTDB:** Bacteria; Proteobacteria; Gammaproteobacteria; Betaproteobacteriales; Rhodocyclaceae; *Dechloromonas*  **NCBI:** Bacteria; Proteobacteria; Betaproteobacteria; Rhodocyclales; Azonexaceae; *Dechloromonas* | 351 | 85 | 7 | 2.90 | 10,315 | *adeF* |
| 9 | **GTDB:** Bacteria; Proteobacteria; Gammaproteobacteria; Enterobacterales; Aeromonadaceae; *Tolumonas*  **NCBI:** Bacteria; Proteobacteria; Gammaproteobacteria; Aeromonadales; Aeromonadaceae; *Tolumonas* | 319 | 85 | 2 | 2.71 | 11,030 | *CRP, qacH* |
| 10 | **GTDB:** Bacteria; Proteobacteria; Alphaproteobacteria; Rhodobacterales; Rhodobacteraceae; *Gemmobacter*  **NCBI:** Bacteria; Proteobacteria; Alphaproteobacteria; Rhodobacterales; Rhodobacteraceae; *Gemmobacter* | 492 | 81 | 5 | 3.05 | 7,144 | *NDM-1,* *floR* |
| 11 | **GTDB:** Bacteria; Proteobacteria; Gammaproteobacteria; Betaproteobacteriales; Burkholderiaceae  **NCBI:** Bacteria; Proteobacteria; Betaproteobacteria; Burkholderiales; Burkholderiaceae | 261 | 95 | 3 | 3.07 | 16,127 |  |
| 12 | **GTDB:** Bacteria; Proteobacteria; Gammaproteobacteria; Betaproteobacteriales; Burkholderiaceae; *Brachymonas*  **NCBI:** Bacteria; Proteobacteria; Betaproteobacteria; Burkholderiales; Comamonadaceae; *Brachymonas* | 204 | 91 | 3 | 1.91 | 12,788 | *OXA-2* |
| 13 | **GTDB:** Bacteria; Proteobacteria; Gammaproteobacteria; Betaproteobacteriales; Burkholderiaceae; *Alicycliphilus*  **NCBI:** Bacteria; Proteobacteria; Betaproteobacteria; Burkholderiales; Comamonadaceae; *Alicycliphilus* | 528 | 80 | 9 | 3.21 | 6,972 |  |
| 14 | **GTDB:** Bacteria; Synergistota; Synergistia; Synergistales; Synergistaceae; *Syner-03*  **NCBI:** Bacteria; Synergistetes; Synergistia; Synergistales; Synergistaceae | 150 | 96 | 3 | 1.83 | 17,824 |  |
| 15 | **GTDB:** Bacteria; Proteobacteria; Gammaproteobacteria; Betaproteobacteriales; Burkholderiaceae  **NCBI:** Bacteria; Proteobacteria; Betaproteobacteria; Burkholderiales; Burkholderiaceae | 145 | 90 | 0 | 2.00 | 18,779 | *mtrR, OXA-21* |
| 16 | **GTDB:** Bacteria; Firmicutes; Clostridia; Saccharofermentanales; Saccharofermentanaceae  **NCBI:** Bacteria; Firmicutes; Clostridia | 31 | 99 | 0 | 1.63 | 104,961 |  |
| 17 | **GTDB:** Bacteria; Actinobacteriota; Actinobacteria; Actinomycetales; Bifidobacteriaceae; *Bifidobacterium; Bifidobacterium longum*  **NCBI:** Bacteria; Actinobacteria; Actinobacteria; Bifidobacteriales; Bifidobacteriaceae; *Bifidobacterium; Bifidobacterium longum* | 240 | 81 | 6 | 1.78 | 9,702 | *ileS* |
| 18 | **GTDB:** Bacteria; Proteobacteria; Gammaproteobacteria; Enterobacterales; Aeromonadaceae; *Tolumonas; Tolumonas auensis*  **NCBI:** Bacteria; Proteobacteria; Gammaproteobacteria; Aeromonadales; Aeromonadaceae; *Tolumonas; Tolumonas auensis* | 179 | 94 | 4 | 2.80 | 23,044 | *CRP, ugd, mdtF* |
| 19 | **GTDB:** Bacteria; Desulfobacterota; Desulfobulbia; Desulfobulbales; Desulfobulbaceae; *Desulfobulbus*  **NCBI:** Bacteria; Proteobacteria; Deltaproteobacteria; Desulfobacterales; Desulfobulbaceae; *Desulfobulbus* | 358 | 96 | 1 | 2.93 | 10,647 |  |
